# A Chemogenomic Toolkit to Evaluate the “Ins and Outs” of Yeast Plasma Membrane Transporters

**DOI:** 10.1128/mbio.00955-22

**Published:** 2022-04-25

**Authors:** Rajendra Prasad, Atanu Banerjee

**Affiliations:** a Amity Institute of Biotechnology, Amity University Haryana, Gurugram, India; b Amity Institute of Integrative Sciences and Health, Amity University Haryana, Gurugram, India

**Keywords:** chemogenomics, *Saccharomyces cerevisiae*, double transporter gene deletion library, drug/xenobiotic transporters, high-throughput screening

## Abstract

Over the years, there has been a lot of emphasis on the development of high-throughput platforms that help identify transporters of drugs and xenobiotics. However, major hinderances in these approaches include substrate promiscuity and functional redundancy of membrane transporters. To tackle such issues, Almeida and colleagues (L. D. Almeida, A. S. F. Silva, D. C. Mota, A. A. Vasconcelos, et al., mBio 12(6):e03221-21, 2021) elegantly used the power of yeast genetics and created a double gene deletion library for 122 nonessential plasma membrane transporters that facilitates high-throughput identification of drug/xenobiotic transporters. While examining a library of cytotoxic compounds, the authors identified a strong correlation between the chemical structure of azoles and possible import/export routes. Interestingly, the authors also identified the *myo*-inositol transporter Itr1 to be responsible for import of triazole and imidazole antifungal compounds and proposed a role for the ABC transporter Pdr5 in carbendazim uptake.

## COMMENTARY

Transporters in the cell membrane are indispensable for the functioning of any organism (1). Clinically speaking, the implications of membrane transporters are immense, as membrane transporters represent the third major target class for drugs listed in the DrugBank database after receptors and enzymes (2). A significant proportion of membrane transporters belonging to the ATP-binding cassette (ABC) superfamily, and the solute transporter proteins (SLC) family, directly interacts with drugs (3). Similarly, the International Transporter Consortium (ITC) emphasizes identification of transporters that are clinically important in drug import and export to help guide preclinical and clinical studies (4). However, a detailed understanding of transporter function and specificity is available for only a small fraction of transporters, partly due to the resource-driven and time-consuming approaches involved. In most cases, a particular compound is transported by multiple transporters, and deletion of a single transporter may not result in an observable phenotype due to the complimentary roles of other transporters. Thus, loss-of-function genetic screens are often inadequate to map drug import and export because of the functional redundancy of membrane transporters. Furthermore, membrane transporters are highly promiscuous. Barring a few exceptions, for the most part, computational approaches have not been successful in providing conclusive molecular descriptors for substrate classifications (5).

The yeast Saccharomyces cerevisiae has been used to study drug import and export (1, 6). It has been employed in yeast chemogenomic studies using haploinsufficiency profiling (HIP) or homozygous deletion profiling (HOP) (7, 8). However, these studies relied on single transporter deletion strains and were not able to detect redundant membrane transporters. Surveys with strains with at least double gene deletions are required to establish relative contributions of multiple transporters to the influx/efflux of drugs.

Given these complexities with membrane transporters, Almeida and colleagues (9) constructed a double-deletion library of 122 nonessential transmembrane transporters in all possible combinations. The double-deletion strains were obtained by carrying out crosses between strains harboring single transporter gene deletions, each carrying a specific replacement cassette, *kan*MX or *nat*MX. Of note, while the *kan*MX cassette carried the flanking barcodes that enable tracking, the *nat*MX was excluded from any such barcodes.

For benchmarking, the resultant library of ~14,000 strains was subjected to toxicity assays using a library of compounds, some of which have been reported to enter the cell using passive diffusion. The authors proposed two complementary approaches for mapping the compound import/export routes, a low-throughput plate-based screen and a high-throughput screen focused on monitoring the abundance of strains in liquid culture.

Through the plate-based screen, the authors observed that certain transporter genes were maximally represented when challenged by specific compounds. For instance, *NHA1* was identified when cells were exposed to ketoconazole (14 hits) and difenoconazole (6 hits), and *FUR4* (11 hits) was identified when cells were exposed to tunicamycin. While overrepresentation of certain genes in the resistant colonies does point to their plausible roles as a transporter for the specific compound, the observation needs further study to eliminate false positives.

**FIG 1:**
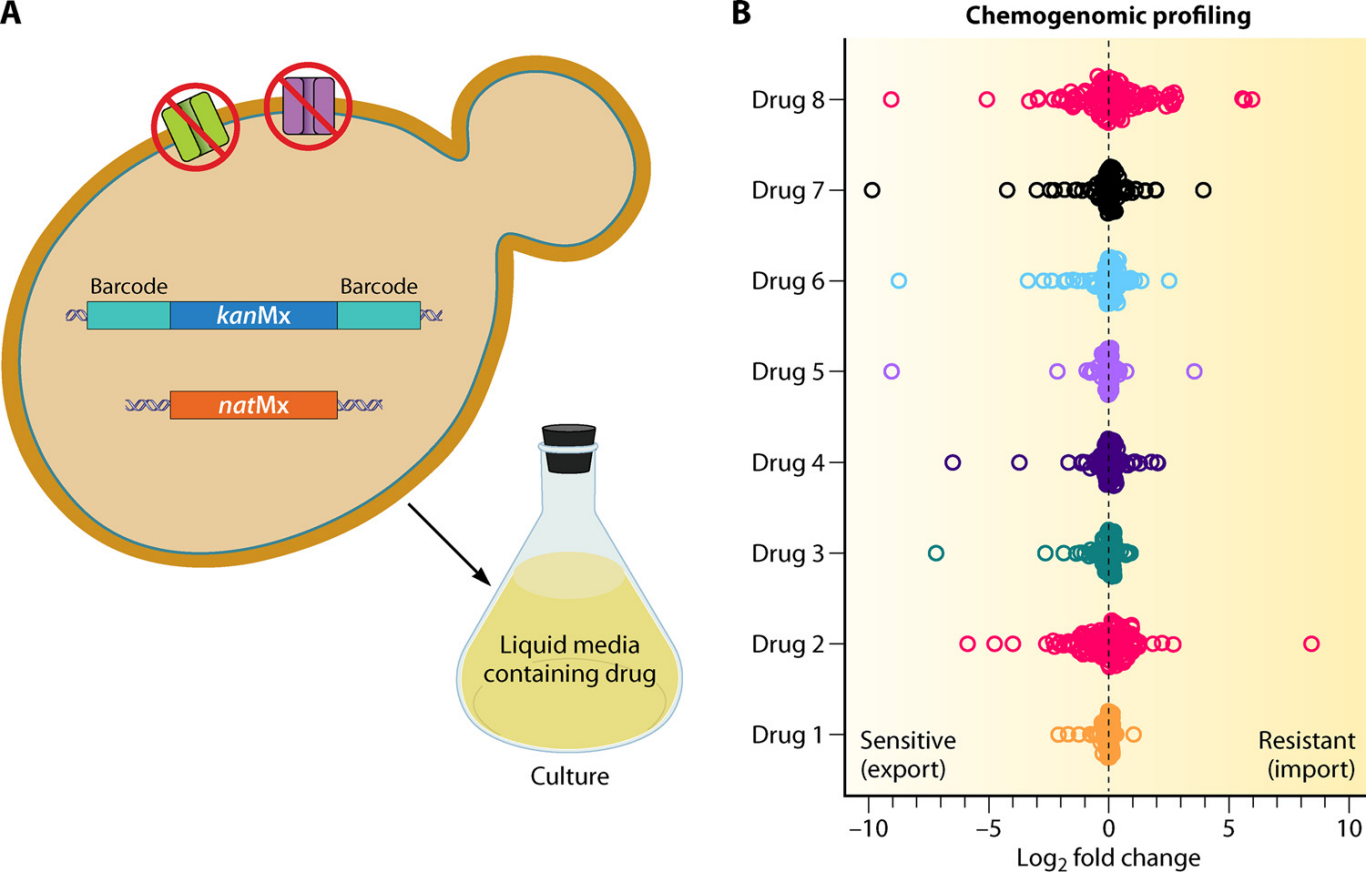
Monitoring import and export routes for drugs via chemogenomic profiling (CGP). (A) The platform comprises of a library of double gene deletions for non-cytoplasmic transporter proteins in all possible combinations. *kan*Mx and *nat*Mx are the two cassettes used for gene deletions. The *kan*Mx cassette is flanked by specific barcodes for gene-sequencing to detect abundance. In this assay, the entire library is cultured in liquid media containing inhibitory concentrations of the drug. (B) The relative abundance of the double-deletion strains is monitored through log_2_ fold change, suggesting a role of the specific transporter genes in import or export functions. While a log_2_ fold change of >0 indicates a plausible role of the transporter protein as an importer, a log_2_ fold change of <0 points to a possible exporter role.

The high-throughput screen (referred to as chemical genomic profiling [CGP]) was proposed as a more robust and sensitive alternative. The double-deletion library is cultured in liquid media with inhibitory concentrations of various xenobiotics, and barcode-sequencing is performed for monitoring abundance of specific-gene deletions within the population. Thus, this platform provides an added advantage of identifying gene deletions that result in susceptibility (probable exporters) and those conferring resistance (probable importers) (Fig. 1). Interestingly, a few prominent exporters could be identified by the authors in these experiments. For instance, gene deletions in ABC transporters *PDR5*, *PDR11*, and *SNQ2* led to susceptibility toward artesunate. Besides artesunate and already established substrates like azoles, *PDR5*Δ led to susceptibility to xenobiotics, such as irgasan and iprobenfos, further expanding the Pdr5 substrate repertoire. Similarly, *YOR1* gene deletion (*YOR1*Δ) conferred susceptibility toward tunicamycin, and *NFT1*Δ conferred susceptibility toward azoles such as epoxiconazole and tebuconazole. Of note, transporters in the major facilitator superfamily (MFS) showed only minor representation as exporters for the test compounds. For instance, while *QDR1*, *QDR2*, and *ATR1* deletions were implicated in 5-fluorocytosine susceptibility, *DTR1* deletion resulted in susceptibility toward fluconazole.

The authors investigated certain drug-transporter relationships in detail. Interestingly, the survey demonstrated strong propensities of certain transporters toward specific chemical signatures in their substrates. For instance, proteins mediating carbendazim transport, such as Tna1, recognize substrates that contain carboxyl group. A much stronger correlation was evident in the case of azole compounds. The agrochemical azoles that are members of 1,2,4-triazole class and harbor a halogenated benzene ring, namely, epoxiconazole, difenoconazole, and tebuconazole, showed similar import and export profiles. The imidazole antifungals ketoconazole and clotrimazole showed a similar behavior in the context of other specific transporters. It is noteworthy that fluconazole did not show any overlap in transporter preferences with other tested classes of azoles, perhaps due to its structural differences. These and other results cast further doubt on the notion that passive diffusion is responsible for uptake of some pharmaceutical drugs, such as fluconazole. Transporter-mediated azole import was an elusive subject until recent studies demonstrated that facilitated diffusion was responsible for azole import in several fungal species (10, 11). Almeida et al. in the current study proposed *myo*-inositol transporter Itr1 (and not its paralog Itr2) as an importer for triazole and imidazole compounds, albeit not an exclusive one. Interestingly, Itr1 was also found to be involved in the import of ketoconazole and clotrimazole in a previous study, which exploited a single gene deletion library performed by the same group (6).

Another interesting finding from this systematic survey includes identification of an import function displayed by many ABC transporters like Yor1, Ybt1, and more surprisingly Pdr5. The authors confirmed that the *PDR5* deletion causes carbendazim (fungicide) resistance, suggesting that it is functioning as an importer. While the majority of the literature discusses the export functions of eukaryotic ABC transporters, recent reports discuss possible import functions (12). The mechanics of Pdr5 support its role as an exporter (13), but its proposed role as importer needs characterization. Thus, the emerging concept of xenobiotic import by eukaryotic ABC transporters needs attention, and further study will be important for identifying their import mechanism.

The double-deletion library constructed in this study proved to be quite efficient in identifying multiple transporters for a single compound and multiple compounds for a single transporter. It will be much more useful if the library was expanded to include the complete yeast plasma membrane transporter armamentarium. The toolkit’s potential could also be maximized if it is tested against a drug/xenobiotic library that includes a wider chemical space. While the approach presented in the study is powerful enough to provide initial leads into the import and export routes for xenobiotics, the results must be complemented and confirmed with *in vitro* transport studies. A significant hit for a gene might not always point to a transport role, and the susceptibility/resistance attribute could be simply due to a marker effect or background mutations in the strain. This was evident in the case of Nha1, which garnered significant hits with ketoconazole in both the plate assay as well as CGP. However, its role as an azole importer could not be established by the authors. Furthermore, susceptibility/resistance may be an indirect effect not directly related to the transporter function but to indirect perturbations in the membrane and/or cellular metabolism/homeostasis (1, 14). Thus, further studies are needed to improve the signal-to-noise ratios by imposing constraints that help exclude false positives as have been described in other chemogenomic profiling studies (8). Existing background strains devoid of multiple membrane transporters, coupled with the additional capability of targeted expression/hyperexpression of transporter candidates, could be used with CGP for further confirmation (15, 16). Lastly, combining genetics with machine learning also offers an attractive option to have a holistic understanding of the transporters mediating the “ins and outs” across the plasma membrane (17, 18).
